# Breast pseudotumoral radionecrosis as a late radiation-induced injury: a case report

**DOI:** 10.1186/1752-1947-3-71

**Published:** 2009-10-08

**Authors:** Holger Gerullis, Christoph Johann Heuck, Paul Schneider

**Affiliations:** 1 Department for General and Thoracic Surgery, DRK Clinics, Drontheimer Strasse, Berlin 13359, Germany; 2 West German Cancer Center (WTZ), University of Essen, 45122 Essen, Germany; 3 Montefiore Medical Center, Department of Medical Oncology, The Albert Einstein Cancer Center, Bronx, NY 10467, USA

## Abstract

**Introduction:**

New therapies and treatment protocols have led to improved survival rates in many cancers. The improved rates are such that patients are now living long enough to experience some negative long-term side effects of the initial therapy.

**Case presentation:**

We report the case of a 65-year-old Caucasian woman who presented with a rare case of pseudotumoral radionecrosis, a late radiation-induced injury, after combined surgical and cobalt radiation therapy for the treatment of adenocarcinoma of the right breast. The patient underwent resection of this benign, yet progressively growing and painful tumor. A cosmetically satisfying result was achieved by reconstruction of the thoracic wall with a polypropylene mesh and a latissimus dorsi muscle flap.

**Conclusion:**

With improved overall survival, new management strategies for late side effects of therapy are becoming of crucial importance for affected patients. In the future, improving toxicity-free survival will be as important as achieving disease-free survival or local tumor control.

## Introduction

With the increasing number of long-term survivors who have been treated with newer anticancer therapy strategies, we encounter increasing numbers of sequelae from earlier therapeutic approaches. These long-term side effects are an important factor in the patient's quality of life, both psychologically and somatically, and must be addressed in the treatment of the underlying disease.

Radiation-induced fibroatrophy (RIF) is a rare late sequela of high-dose radiation therapy. It is localized to the radiation field and is usually irreversible. The pathologic manifestation of this entity can be limited to skin dryness, hyperpigmentation or telangiectasia. This superficial damage, however, may also be combined with underlying fibronecrotic lesions affecting the pleura and lungs resulting in pulmonary fibrosis, neurological disorders secondary to the development of perineural fibrosis, or spontaneous rib fractures due to osteoradionecrosis [1-3]. We report the excellent cosmetic and functional results after surgical treatment in a particular clinical setting of pseudotumoral radionecrosis as a late radiation-induced injury after initial anticancer treatment.

## Case presentation

**Figure 1:**
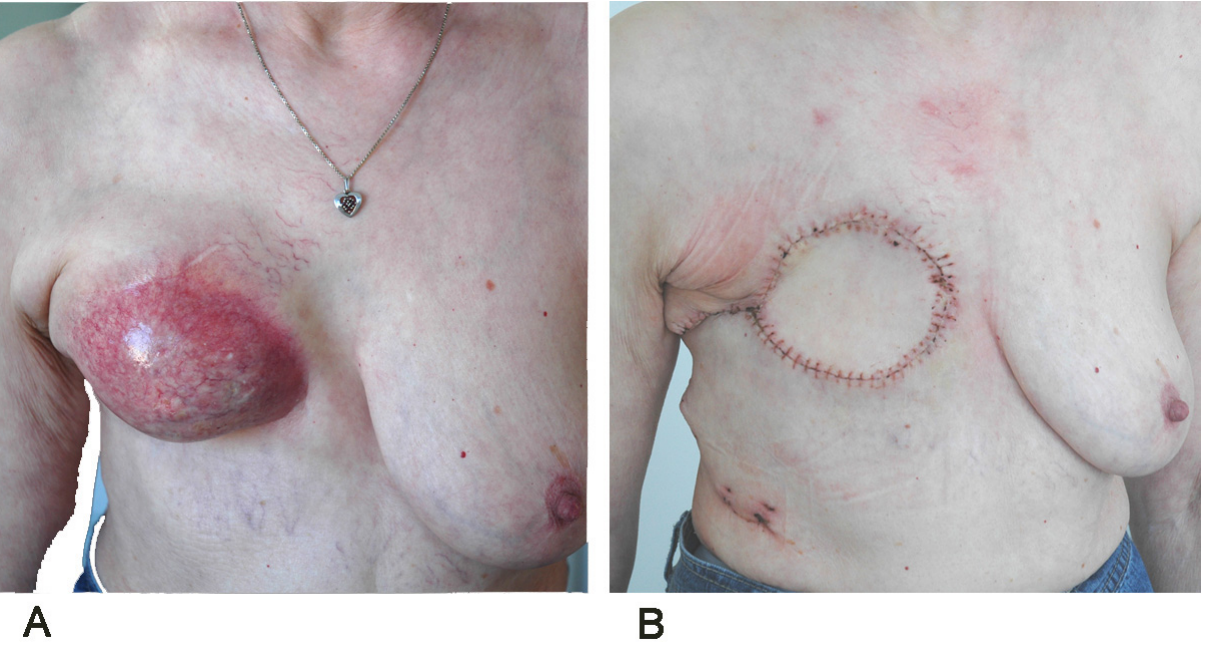
**Pre- and postoperative macroscopic presentation**. (A) Calcific subcutaneous, painful, progressively growing tumor on the right breast 23 years after 60 Gy cobalt radiation before the corrective operation. (B) Post-corrective surgery: after reconstruction of the thoracic wall with a double layer polypropylene mesh and switching of a flap of latissimus dorsi muscle to the thoracic wall.

We report the case of a 65-year-old Caucasian woman who presented with a subcutaneous inflammatory tumor of the right breast, which had been progressively growing over a period of 10 years (Figure 1). The patient had a past history of a multifocal adenocarcinoma of the right breast, which was resected 23 years earlier. Further treatment included chemotherapy with six cycles of fluorouracil, doxorubicin and cyclophosphamide in combination with cobalt radiation with a total dose of 60 Gy, followed by tamoxifen for a total of 12 years. The first local signs were observed seven years after the radiation treatment with the loss of irradiated skin elasticity followed by increasing induration and telangiectasia. Over the 10 years before presentation, the mass had significantly increased in size and become increasingly painful, restricting the patient psychologically and functionally in her daily activities. The patient's other past medical history included high blood pressure and hypercholesterolemia. Her only additional surgery was an appendectomy during childhood.

**Figure 2:**
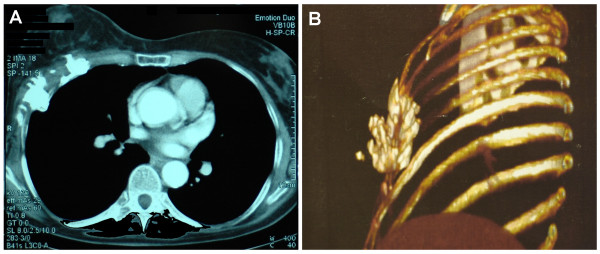
**Pre-operative imaging studies of the tumor**. Computed tomographic scan of the thorax (A) and respective 3-D image (B) exhibiting a large area of costal and muscular radionecrosis. The pleura was not affected.

**Figure 3:**
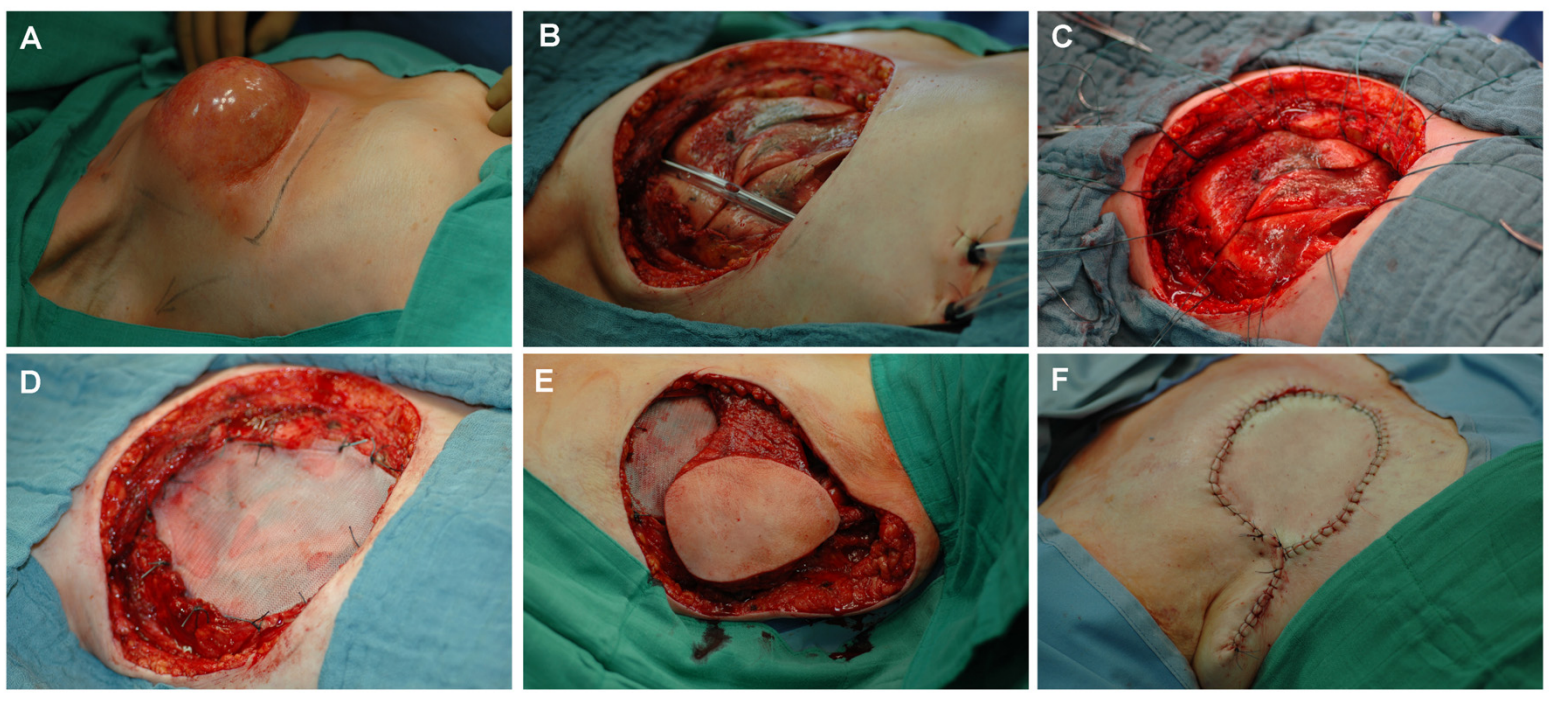
**Intra-operative situs and steps in the surgical procedure**. (A) Operative field; (B) placement of two thoracic drains after resection of the tumor; (C, D) reconstruction of the thoracic wall with a double layer polypropylene mesh; (E) mobilization and switching of the latissimus dorsi flap to the thoracic wall; (F) wound closure.

Diagnostic work-up (bone scintigraphy, abdominal ultrasound, thoracic computed tomography (CT) scan) revealed no local or distant metastases (Figure 2). As there was no suspicion for a progressive malignant disease, we did not perform a pre-operative biopsy. We offered the patient resection of the thoracic wall, reconstruction by a Premilene^® ^mesh and switching of an ipsilateral latissimus dorsi muscle flap. Intra-operatively, we observed multiple retromammarial adhesions without macroscopic tumor infiltration. Pathological fractures of costae 3/4/5 were also observed. The tumor could be removed in its entirety. Reconstruction of the thoracic wall was performed with a double layer polypropylene mesh (Premilene^®^, Braun, Melsungen), fixed with Ethibond^® ^(Ethicon, Norderstedt) sutures. A flap of the latissimus dorsi muscle was then mobilized and switched to the thoracic wall. The muscle was fixed circularly, subcutaneously and cutaneously. Two thoracic drains were placed (Figure 3). The whole operation time was 210 minutes, and blood loss remained minimal at 50 ml.

**Figure 4:**
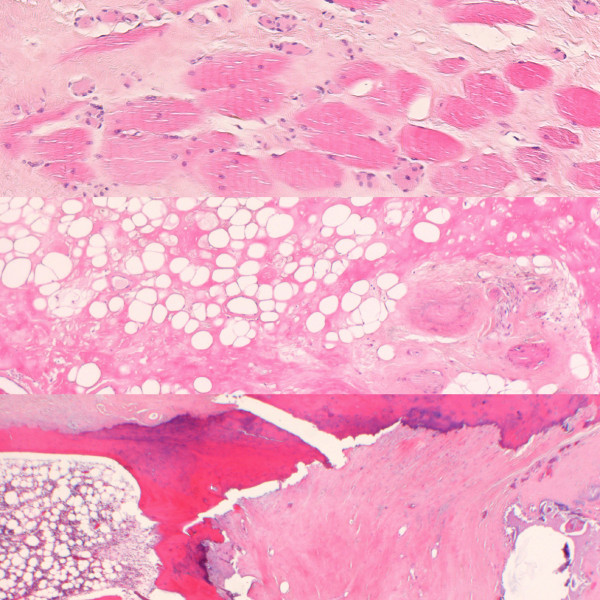
**Histological characteristics of the specimen**. Wide, almost scarred, radionecrosis and fibrosis of muscle, fat and bone, and extended dystrophic calcifications corresponding to a radiation-induced vasculopathy.

The thoracic drains were removed on postoperative days 4 and 8, and the patient left our institution on postoperative day 18. Microscopic examination of the resected specimen showed a wide, almost scarred, radionecrosis and fibrosis of fat, muscle and bone (Figure 4). Also noted were extended dystrophic calcifications corresponding to a radiation-induced vasculopathy. Histologically, there was no evidence of malignancy. Within a follow-up of 14 months, the cosmetic result remained satisfactory without any disturbance in healing.

## Discussion

In the majority of cases, cancer is an aggressive disease which is often fatal and requires consequential therapeutic approaches with agents that provide a narrow therapeutic window. The toxicity of the therapy and the acceptable risk of complications have to be balanced against the risk of recurrence of the malignancy. Radiation therapy has been one of the pillars of cancer therapy for many years. Many technological advances have been made to improve radiation delivery to the targeted area while reducing damage to the surrounding normal tissue. However, with improving survival of our patients, we are now able to observe the long-term effects of earlier therapies, which, at the time they were applied, were in accordance with the best knowledge available.

The spectrum of late radiation-induced injuries is quite heterogeneous but the injuries are usually benign. They may manifest themselves as breast edema, fibrosis, telangiectasia, pulmonary fibrosis, chronic ulceration, rib fractures, fat necrosis or osteonecrosis [4]. The main differential diagnosis is malignant radiation-induced sarcoma (RIS), which has a very poor prognosis [5].

Fajardo differentiates between epithelial and/or parenchymal, stromal and vascular lesions as delayed injuries from ionizing radiation [6]. Epithelial and/or parenchymal lesions include atrophy, necrosis, metaplasia, cellular atypia, dysplasia, and neoplasia. Fibrosis, fibrinous exudates, necrosis and atypical fibroblasts are considered to be common stromal lesions. Vascular lesions affect the microvessels producing lethal and sublethal damage to the endothelial cells, with capillary rupture or thrombosis, and may present as telangiectasia [6].

These changes may occur in any organ. The incidences of complications involving tissues that have a slow turnover increase with time after radiation while most acute reactions are observed in epithelial tissues. Therefore, a long follow-up is necessary in order to fully understand the toxicity of the treatment. The increasing number of long-term survivors allows us to observe these important late side effects more frequently.

In the future, the ability to provide toxicity-free survival may be as important an outcome as achieving a disease-free survival or local tumor control [7]. Perception of a long-term injury from prior treatment as a burden may be very different for the patient and the doctor, in particular, if the morbidity is irreversible, protracted, uncontrollable, painful or socially disabling.

In order to evaluate the most effective therapeutic strategy and to permit quantification of late effects on normal tissue, all patients should be systematically monitored using a scoring system such as the LENT-SOMA (late effects normal tissues - subjective, objective, management, and analytic) scale, as proposed by The European Organization for Research and Treatment of Cancer and the Radiation Therapy Oncology Group (EORTC/RTOG) [5,7,8].

A variety of conservative strategies have been evaluated in order to manage radiation-induced fibroatrophic processes. Treatment approaches include superoxide dismutase or combined pentoxifylline-tocopherol application, underlining the importance of an antioxidant pathway [9,10]. However, in our particular case, surgery was unavoidable. As the possibility of local recurrence or persistence of the primary malignant tumor within the radiated tissue exists, an adequate pre-operative diagnostic work-up must be done before planning a resection and/or reconstructive surgical strategy [11,12].

We report the excellent surgical result of performing an ablatio mammae with resection of the thoracic wall, and reconstruction with a synthetic mesh and a latissimus dorsi muscle flap.

In our patient, the symptomatic pain in the right chest decreased minimally postoperatively. This may have resulted from the surgical trauma after the radical surgery, which was performed in order to reduce as much of the irradiated tissue as possible. Subjectively, the patient reported high satisfaction with the cosmetic and functional outcome. This corresponds to the reported phenomenon that patients evaluate operative results less critically [13,14].

## Conclusion

Radiation-induced long-term side effects may have a benign or malign nature. The aggressive and poor prognostic nature of RIS is the main malignant differential diagnosis for late radiation-induced alterations. Late radiation-induced fibroatrophy (RIF) as a benign adverse event is a rare, irreversible and unavoidable damage after radiotherapy. It may adversely affect the functional and esthetic outcome of patients. Besides conservative treatment options including superoxide dismutase or combined pentoxifylline-tocopherol, secondary radical surgery with exeresis of the necrotic defect and flap reconstruction is the usual treatment in the case of combined superficial RIF with spontaneous or trauma-induced fibronecrotic lesions of the underlying irradiated tissue and bones. It permits excellent cosmetic and functional results.

## Abbreviations

CT: computed tomography; EORTC: European Organization for Research and Treatment of Cancer; LENT: late effects normal tissues; RTOG: Radiation Therapy Oncology Group; RIS: radiation-induced sarcoma; RIF: radiation-induced fibroatrophic process; SOMA: subjective, objective, management, and analytic

## Consent

Written informed consent was obtained from the patient for publication of this case report and any accompanying images. A copy of the written consent is available for review by the Editor-in-Chief of this journal.

## Competing interests

The authors declare that they have no competing interests.

## Authors' contributions

HG drafted the manuscript, performed the literature review and participated in the surgery. PS performed the surgery and supervised the preparation of this report. CJH assisted in the preparation of this manuscript and in the literature review. All authors read and approved the final manuscript.
